# Factors influencing older adults’ adoption of AI voice assistants: extending the UTAUT model

**DOI:** 10.3389/fpsyg.2025.1618689

**Published:** 2025-11-10

**Authors:** Haoran Li, Xin Wei

**Affiliations:** 1 Department of Philosophy, Autonomous University of Barcelona, Barcelona, Spain; 2 Faculty of Geography and History, University of Barcelona, Barcelona, Spain

**Keywords:** elderly users, AI voice assistants, technology acceptance, UTAUT model, user experience

## Abstract

**Introduction:**

With the acceleration of global population aging and the digitalization process, the potential application of AI voice assistants among the elderly has become increasingly apparent. However, the adoption of this technology by older adults remains relatively low. Based on the Unified Theory of Acceptance and Use of Technology (UTAUT), this study extends the model by introducing two variables, perceived AI experience and perceived AI trustworthiness, to explore the key factors influencing older adults’ use of AI voice assistants.

**Methods:**

Data were collected through a structured survey, with participants consisting of 413 elderly users from Shanxi Province, China, using a convenience sampling method. The gender distribution was 53.1% male and 46.9% female, with ages ranging from 60 to 75 years and older. The data were analyzed using Structural Equation Modeling (SEM).

**Results:**

The results showed that performance expectancy, facilitating conditions, perceived AI trustworthiness, and perceived AI experience all had a significant positive effect on the elderly’s intention to use AI voice assistants, while effort expectancy negatively influenced the intention. Additionally, although social influence significantly affected perceived AI trustworthiness, its impact on the intention to use was not significant. Furthermore, intention to use played an important mediating role in the actual behavior of older adults using AI voice assistants.

**Discussion:**

This study enriches the application of the UTAUT model in technology adoption research among older populations by incorporating perceived AI experience and perceived AI trustworthiness. The findings provide practical guidance for optimizing the design and promotion strategies of age-friendly AI voice assistants, highlighting the importance of enhancing user trust and experience to improve technology adoption among the elderly.

## 1 Introduction

With the accelerating progression of global population aging and digitalization, contemporary society faces unprecedented challenges (Liu and McKibbin, 2022). According to projections by the World Health Organization (WHO), the global population aged 60 and above is expected to nearly double by 2050, reaching approximately 2.1billion and accounting for 16% of the global population (Banke-Thomas et al., 2020; Officer et al., 2016; Zhang et al., 2025). This demographic shift will have profound implications for social, economic, and healthcare systems, particularly in the context of aging in place. The WHO Global Age-friendly Cities Guide (2007) outlines eight essential domains for age-friendly environments: (1) outdoor spaces and buildings, (2) transportation, (3) housing, (4) social participation, (5) respect and social inclusion, (6) civic participation and employment, (7) communication and information, and (8) community support and health services (WHO, 2007). As a powerful embodiment of modern information technology, digital technologies hold significant potential to support older adults by enhancing health monitoring, promoting independence, and reducing feelings of loneliness (Boström et al., 2022; Jnr, 2024). However, the digital divide and the challenge of technology adaptation remain critical issues to be addressed (Czaja and Ceruso, 2022; McDaid and Park, 2024). Consequently, in the dual context of aging and digital transformation, how to innovatively integrate technology and services to enhance the autonomy and quality of life of older adults has become a globally pressing concern.

AI voice assistants have become increasingly important in the lives of older adults, facilitating tasks such as communication, health management, and social interaction (Boot, 2022; Lawson, 2023). Prior studies have shown that AI voice assistants can support older users in various aspects, including using smartphones more easily, shopping, learning, entertainment, and social interaction. Through natural language processing technologies, these assistants enable voice-controlled operations—ranging from making calls to controlling appliances, setting reminders, and checking the weather—which not only enhance the convenience of human-computer interaction but also reduce the reliance on traditional input devices such as keyboards and mice. This is especially valuable for older adults with visual or mobility impairments (Cao et al., 2024; Sen, 2023; Thakur and Varma, 2023). Furthermore, voice assistants can aid in daily health monitoring tasks, such as health tracking, medication management, and dietary planning, and may even provide emergency calling functions in critical situations, thereby offering a greater sense of security (Guerreiro and Loureiro, 2023).

Despite the benefits, adoption of AI voice assistants remains low among older adults, primarily due to concerns about privacy, security, and unfamiliarity with the technology (Cao et al., 2024; Zhong et al., 2024).

This study adopts the Unified Theory of Acceptance and Use of Technology (UTAUT) framework to explore the key factors influencing elderly individuals’ acceptance and usage of AI voice assistants. According to the UTAUT model, technology acceptance and use are determined by four main factors: performance expectancy, effort expectancy, social influence, and facilitating conditions (Akdim and Casaló, 2023; Kernan Freire et al., 2023). First, older adults’ expectations that AI voice assistants can improve quality of life and health management, which refers to performance expectancy, play a significant role in their acceptance. Second, the perceived ease of use, or effort expectancy, is especially crucial for this demographic. Social influence reflects the extent to which support from family and friends can positively impact technology acceptance, particularly when these individuals have prior experience with the technology. Finally, facilitating conditions refer to whether older adults have the necessary resources such as devices, internet connectivity, and learning support; the lack of such conditions can significantly reduce their willingness to adopt the technology. Compared with earlier studies, recent developments in UTAUT emphasize the importance of social support and psychological factors in influencing elderly users’ acceptance of technology, particularly in populations where the digital divide is pronounced (Wang et al., 2024; Yu and Chen, 2024). As technology and society evolve, changes in performance expectancy, effort expectancy, social influence, and facilitating conditions have led to a greater willingness among older adults to embrace new technologies.

Moreover, perceived trust and user experience are also critical determinants of elderly users’ acceptance of AI voice assistants. Older adults typically exhibit lower levels of trust in AI technologies than younger users, especially with regard to privacy and data security concerns. Research has shown that when older adults perceive that the system can provide accurate, reliable information while safeguarding their privacy, they are more likely to adopt it (Thakur and Varma, 2023; Sen et al., 2023). Additionally, factors related to user experience, such as ease of use, system responsiveness, and the accuracy of speech recognition, directly affect whether older adults are willing to continue using such technologies (Yu and Chen, 2024).

By identifying and analyzing the key factors affecting older adults’ acceptance of AI voice assistants, this study aims to provide both theoretical insights and practical guidance for promoting the widespread adoption of this technology among the elderly. Rooted in the UTAUT framework and supplemented by dimensions such as perceived trust and experience, this research offers a comprehensive theoretical model to understand how older users adopt and continue using AI voice assistants. In particular, this study is the first to integrate perceived AI experience and perceived AI trustworthiness into the UTAUT model to explore the deeper psychological mechanisms underlying older adults’ technology adoption. From a practical perspective, this study also proposes feasible strategies to enhance trust and usability for older adults, contributing to improved health management, autonomy, and quality of life in aging-in-place contexts. Ultimately, the findings will support the application of AI technologies among elderly populations and promote greater social participation and life satisfaction, thereby offering significant societal and practical value.

## 2 Literature review

### 2.1 Artificial intelligence voice assistants

AI voice assistants are software applications that utilize artificial intelligence technologies such as machine learning, natural language processing, and speech recognition to interact with users through voice commands. They offer personalized responses, task management, and information retrieval (Gupta and Nagar, 2024; Kumar 2024; Yadav et al., 2023). First introduced by IBM’s Watson system in 2011, voice assistants such as Amazon Alexa, Google Assistant, and Apple Siri have since evolved to perform a wide range of tasks, including making calls, scheduling appointments, and managing user preferences (Nasirian et al., 2017; Pakhmode et al., 2023; Dev et al., 2025; Mahesh, 2023). These advancements have made AI assistants increasingly valuable, particularly for older adults, by enabling them to interact with technology more easily, even in the presence of cognitive or physical impairments (Liu et al., 2022; Baumann et al., 2025).

While the technology has made significant progress, including improved speech recognition accuracy and personalized customization, challenges still exist, especially for elderly users with cognitive impairments or hearing issues. Despite these improvements, older adults continue to face difficulties in adopting and using these assistants, which highlights the need to overcome various technological barriers (Le Pailleur et al., 2020). The widespread acceptance of AI voice assistants among older adults remains limited, often hindered by unfamiliarity, privacy concerns, and a lack of trust in AI systems (Cao et al., 2024; Zhong et al., 2024). As a result, examining factors that influence the acceptance and use of AI assistants by older adults remains an essential area of research (Paringe et al., 2023).

### 2.2 Current research on older adults’ adoption of AI voice assistants

In recent years, research on older adults’ use of AI voice assistants has developed rapidly. Current studies on AI voice assistants mainly focus on three areas: (1) voice technology, (2) voice user experience, and (3) user adoption intention (Karkera et al., 2023; Zhong et al., 2024).

In terms of voice technology, research related to older adults’ use of AI voice assistants focuses on improving the adaptability of speech recognition, semantic understanding, and speech synthesis to better meet the needs of the elderly (Elghaish et al., 2022). Given that older adults may face issues such as unclear speech or non-standard pronunciation, researchers have employed enhanced speech recognition technologies, such as deep learning-based CNN and RNN models, to improve recognition accuracy under different accents, speech speeds, and noisy environments (Qian and Honggai, 2023; Subhash et al., 2020). Moreover, to better understand commands given by older adults—especially when dialects or non-standard expressions are used—researchers have optimized semantic understanding using natural language processing techniques (Pakhmode et al., 2023). To enhance elderly users’ acceptance of voice assistants, modifications have also been made in speech synthesis by applying more human-friendly voice models, such as WaveNet and Tacotron, aiming to deliver more natural and understandable voice outputs, thereby reducing cognitive load and emotional barriers during use.

Regarding voice user experience, studies emphasize the interaction experience of older adults with voice assistants, particularly the integration of technical performance and emotional factors. Research shows that older adults prefer voice assistants that respond quickly, provide clear speech, and offer stable feedback (Kiseleva et al., 2016). Additionally, the interface design should be simple and intuitive, and the interaction should be natural and smooth. This not only reduces operational complexity for older users but also enhances their confidence and comfort during use (Cohen et al., 2004; Blit-Cohen and Litwin, 2004). At the same time, emotional design plays a crucial role—warm and friendly voice feedback can significantly improve older adults’ willingness to use voice assistants and their overall satisfaction, making the interaction experience more comfortable and pleasant.

As for user adoption intention, research mainly explores older adults’ acceptance and willingness to use voice assistants. Older adults may face various challenges when using new technologies, such as technological barriers, cognitive burdens, and psychological resistance (Davis, 1989). Consequently, researchers often use the Technology Acceptance Model (TAM) and the extended Unified Theory of Acceptance and Use of Technology (UTAUT) to analyze the factors influencing older adults’ adoption of voice assistants (Venkatesh et al., 2003). Findings indicate that perceived usefulness, perceived ease of use, and social influence are key factors affecting adoption (Cao et al., 2024). In addition, older adults’ educational background, technological proficiency, and social support also have significant effects on their intention to adopt (Liu et al., 2023).

In summary, speech technology, user experience, and adoption intention are three critical dimensions in the study of older adults’ use of AI voice assistants. By continuously optimizing speech technologies, enhancing user experience, and improving older adults’ willingness to adopt, the use of AI voice assistants among this demographic can be further promoted, ultimately improving their quality of life.

### 2.3 UTAUT model and older adults’ technology use

**Figure 1:**
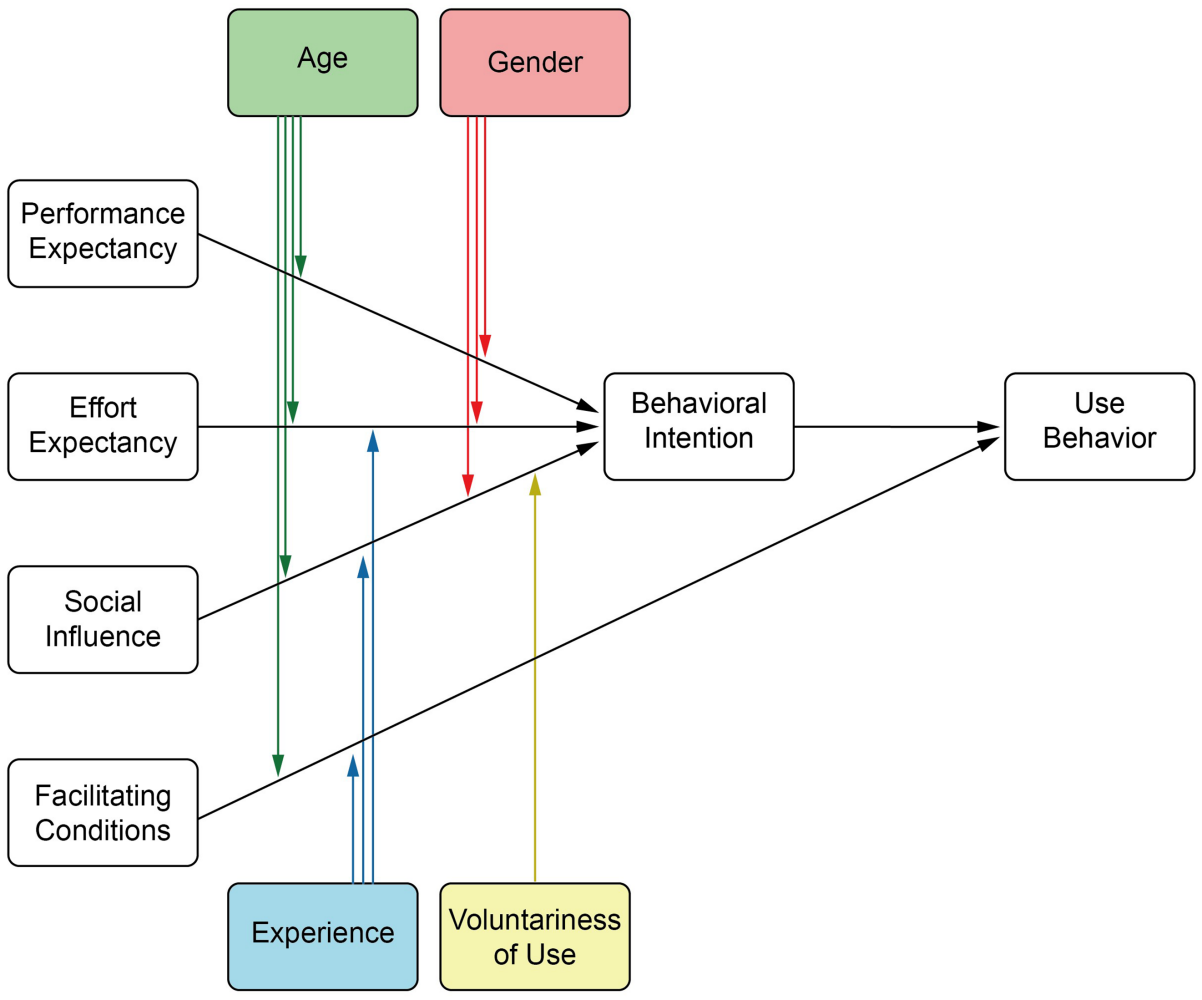
Unified theory of acceptance and use of technology (UTAUT).

The UTAUT model is a useful theoretical framework for studying individual acceptance and use of new technologies (Khechine et al., 2016) (see Figure 1). When studying older adults’ use of AI voice assistants, it is essential to apply the UTAUT model while considering the characteristics and differences between traditional and modern voice assistants. Compared to traditional voice assistants, current AI voice assistants have significantly improved in terms of accuracy and naturalness in speech recognition, semantic understanding, and speech synthesis. As a result, older adults’ perceived AI experience has improved, leading to changes in factors such as performance expectancy, effort expectancy, social influence, and facilitating conditions when using modern AI voice assistants (Joshi, 2025).

In the UTAUT model, performance expectancy refers to the degree to which individuals believe that using new technology will improve their task performance and efficiency. In the context of older adults using AI voice assistants, performance expectancy is reflected in their perception and expectation that voice assistants can help with daily tasks, provide convenience, and enhance quality of life. Due to significant advancements in speech recognition and semantic understanding, older adults can now manage everyday affairs more easily, such as health monitoring and voice-controlled home appliances. Therefore, their performance expectancy has increased, and modern AI voice assistants offer greater support and potential than their traditional counterparts (Lee et al., 2025; Yu and Chen, 2024).

Effort expectancy refers to the perceived cognitive load and difficulty associated with learning and using new technology. For older adults, this primarily involves their perception of the mental effort, required skills, and learning cost involved in operating AI voice assistants. With the increasing accessibility and user-friendly design of modern AI voice assistants, simpler voice commands and intelligent feedback have reduced the cognitive demands and learning complexity (Ammenwerth, 2019; Li, 2025). In comparison to traditional models, the optimized design of modern AI voice assistants facilitates easier learning and operation for older users, reducing cognitive load and thus lowering effort expectancy (Rouidi et al., 2022; Venkatesh, 2022).

Social influence refers to the extent to which family, friends, or community members influence older adults’ attitudes and behaviors toward adopting AI voice assistants. This reflects the social support and encouragement older adults receive from their social network. In the case of older adults, social influence is mainly manifested in the support and encouragement received from family, friends, and community when using AI voice assistants (Joa and Magsamen-Conrad, 2022; Zhou et al., 2019). As societal acceptance of smart devices increases, older adults are more likely to receive positive reinforcement from those around them. The intelligence and convenience of modern AI voice assistants bring significant improvements to older adults’ lives, such as home automation control and medication reminders, which further strengthen social support and influence (Wang et al., 2020; Xu et al., 2022).

Facilitating conditions refer to the degree to which individuals perceive the availability of the infrastructure and support necessary to use new technology (Zhou et al., 2019). For older adults, this includes internet connectivity, device availability, and technical support. With significant progress in technical support and infrastructure, the proliferation of 5G and smart home technologies has made it easier for older adults to access the necessary resources and assistance. Compared to traditional voice assistants, modern AI voice assistants provide stronger technical support and more stable performance, greatly enhancing older adults’ acceptance and willingness to use new technologies (Lee et al., 2025).

Based on the above, this study proposes the following hypotheses:

*H1*: Facilitating conditions positively influences older adults' intention to use AI voice assistants.

*H2*: Performance expectancy positively influences older adults' intention to use AI voice assistants.

*H3*: Effort expectancy positively influences older adults' intention to use AI voice assistants.

*H4*: Social influence positively influence older adults' intention to use AI voice assistants.

### 2.4 Perceived AI experience

The experience of older adults using AI voice assistants is influenced by various factors, among which perceived AI experience plays a critical role. Perceived AI experience refers to the quality of interaction that older adults experience with AI voice assistants, including the smoothness of interaction, accuracy of speech recognition, and system responsiveness. This construct focuses on the user’s perception of the system’s ability to understand and process commands effectively, and the overall ease and comfort of use (Cheng and Jiang, 2020; Hwang and Won, 2022). In the past, traditional voice assistants mainly relied on preset voice commands and fixed responses, resulting in relatively limited and non-personalized interaction processes, which weakened the overall user experience. With the rapid advancement of AI technologies, modern voice assistants have significantly improved in terms of accuracy and naturalness in speech recognition, semantic understanding, and speech synthesis. Interactions between users and voice assistants have become more fluid and intelligent, leading to a substantial enhancement in perceived AI experience when using modern AI voice assistants (Kiwa et al., 2024; Jnr, 2024; Lawson, 2023).

Older adults’ perceived AI experience plays a key role in their decision-making process. When they notice significant improvements in speech recognition accuracy, naturalness of semantic understanding, and fluency of speech synthesis, they also perceive improvements in the infrastructure and support required to use these technologies (Lopatovska, 2020). This enhanced perception not only increases their acceptance of AI voice assistants but also strengthens their awareness of the facilitating conditions needed. Older adults begin to believe that the necessary hardware (such as device availability and network connectivity) and social support (such as help from family or the community) have also been effectively enhanced. Thus, the improvement in perceived AI experience positively influences their perception of facilitating conditions (Qiu and Ishak, 2025; Tu et al., 2023), meaning they are more likely to seek environments and resources that provide robust technical and social support, laying the foundation for wider adoption of AI voice assistants.

In addition, as the performance of AI voice assistants improves, older adults’ expectations for convenience also increase. They hope that voice assistants can help them complete daily tasks more efficiently, such as setting reminders, retrieving information, or managing smart home devices (Zhang et al., 2025). When they perceive that AI voice assistants can improve their quality of life and convenience, their performance expectancy also increases (Boström et al., 2022; Liu et al., 2022). Modern voice assistants not only offer higher accuracy but also provide a smoother and more natural interaction experience, leading older adults to believe these assistants can bring more tangible benefits, thereby raising their expectations of the technology’s performance.

Perceived AI experience is negatively correlated with effort expectancy. With more intuitive operations and quicker responses, older adults no longer need to invest significant time and energy to learn complex procedures. Simple user interfaces and fast feedback allow them to quickly adapt to the technology, reducing their psychological burden associated with learning and usage (Liu et al., 2024). Simple user interfaces and fast feedback allow them to quickly adapt to the technology, reducing their psychological burden associated with learning and usage (Guerreiro and Loureiro, 2023; Xiao and Boschma, 2023). This perceived simplicity and ease of use significantly lower their expectations regarding operational difficulty, indicating a negative relationship between perceived AI experience and effort expectancy.

In summary, older adults’ perceived AI experience influences their technology acceptance and usage intention on multiple levels. An enhanced perceived AI experience is positively correlated with their expectations of technical support and infrastructure, their perception of technological performance, and negatively correlated with their expected difficulty and learning cost. These factors collectively contribute to the broader acceptance and long-term use of AI voice assistants among older adults.

Accordingly, this study proposes the following hypotheses:

*H5-a*: Perceived AI experience positively influences facilitating conditions.

*H5-b*: Perceived AI experience positively influences performance expectancy.

*H5-c*: Perceived AI experience positively influences effort expectancy.

### 2.5 Perceived trust in AI

Perceived trust refers to the extent to which users trust a product or service in terms of its reliability, security, ease of use, human-centered design, adaptability, and privacy protection (Cao et al., 2024; Song and Luximon, 2020; Wischnewski et al., 2024; Ziefle and Arning, 2018). In the past, traditional voice assistants relied on preset commands and responses and lacked personalized services, resulting in relatively low levels of user trust. However, with advances in AI technology, modern voice assistants can now provide more personalized and natural interactions, which has strengthened users’ sense of trust. The establishment of perceived trust relies not only on technical accuracy and reliability but also on system transparency and explainability (Simuni, 2024; Balasubramaniam et al., 2023). For users, trust should be built through a transparent process, where they can understand how and why the system generates specific responses or content, thus helping to foster confidence in the system (Bisconti et al., 2024; Pataranutaporn et al., 2023).

Social influence plays an important role in shaping older adults’ perceived trust in AI (Riyanto and Jonathan, 2018). Older adults often rely on the opinions and support of family, friends, and community members when making decisions, especially regarding the adoption of new technologies. Social support helps older adults perceive technologies as more reliable and secure (Corrigan et al., 1980; Le et al., 2024). When family or friends recommend or use AI voice assistants themselves, older adults are more likely to view the technology as trustworthy, increasing their own level of trust. For example, when children or friends demonstrate the convenience of AI voice assistants in everyday life, this social influence can reduce older adults’ fear and uncertainty about technology and enhance their trust in AI voice assistants (Sun, 2025). Therefore, social influence serves as a positive driver by strengthening trust in the technology among older adults.

Perceived trust in AI is associated with older adults’ willingness to use AI voice assistants. When older adults have a high level of trust in AI voice assistants, they are more likely to accept and adopt the technology (Kraus et al., 2024). Trust encompasses several aspects, including technological reliability, security, privacy protection, as well as the assistant’s ability to offer personalized and adaptive services. When older adults believe that a voice assistant is secure, protects their privacy, and can adapt to their needs, they feel more at ease and comfortable, which increases their willingness to use it. Research has shown that users with high trust in a technology are more likely to use it consistently over time, as trust reduces concerns about technical failures or privacy breaches and enhances motivation for continued use (Gedrimiene et al., 2023). Therefore, perceived trust in AI has a direct and positive effect on older adults’ willingness to use AI voice assistants.

Accordingly, this study proposes the following hypotheses:

*H6*: Social influence positively affects older adults' perceived trust in AI.

*H7*: Perceived trust in AI positively affects older adults' intention to use AI (voice) assistants.

*H8*: Older adults' intention to use AI (voice) assistants positively affects their usage behavior.

## 3 Research methodology

### 3.1 Research model

**Figure 2:**
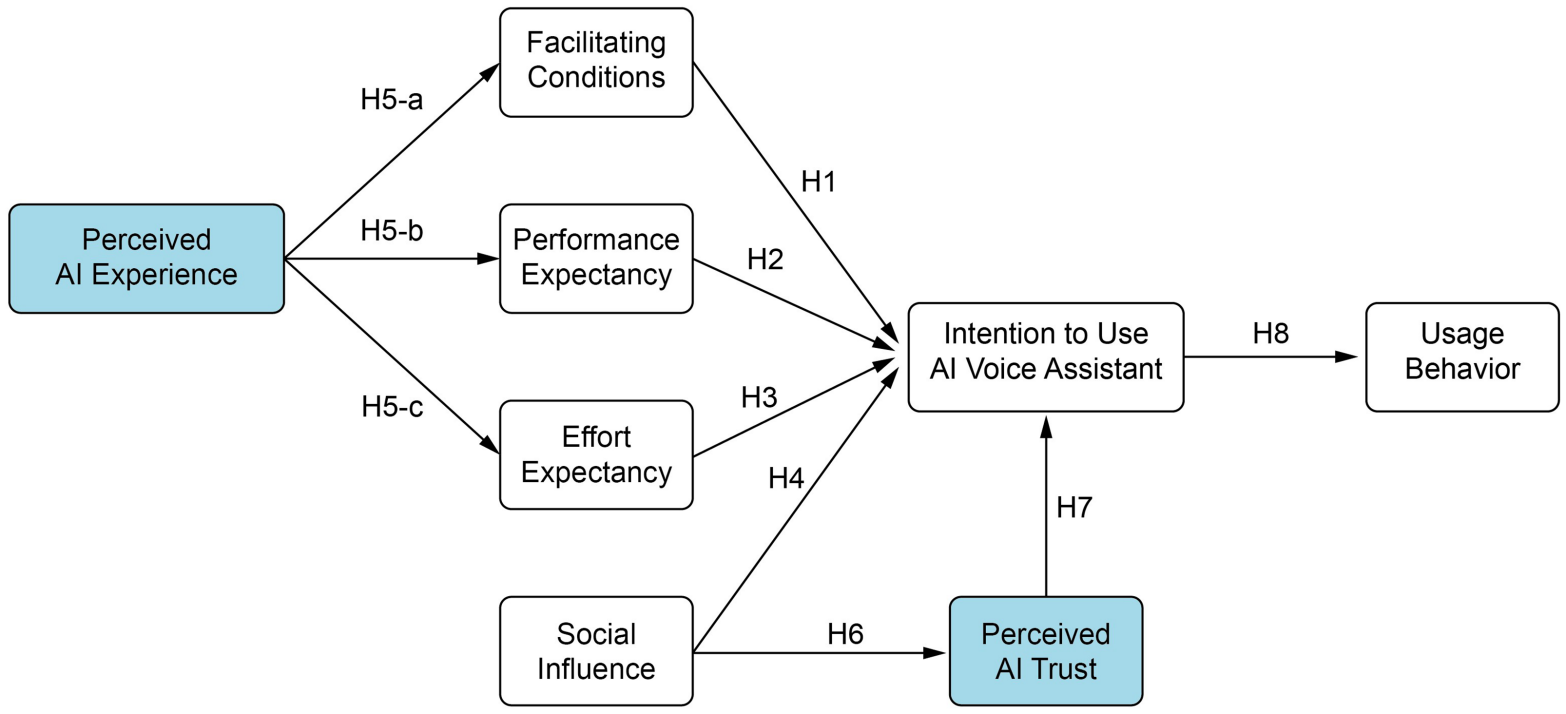
Research model.

Based on the hypotheses proposed above, this study presents the following research model (see Figure 2).

### 3.2 Definition and measurement of variables

The definitions, codes, measurement items, and corresponding scale sources for all variables in this study are detailed in Table 1. All items are measured using a 5-point Likert scale (1 = strongly disagree, 5 = strongly agree). By integrating multiple validated scales, this study effectively measures the specific performance of each variable, ensuring the scientific rigor and reliability of the research results.

**Table 1:** Definitions, codes, measurement items, and referenced scales for all variables.

Research variable	Operational definition	Code	Item	Source
Perceived AI trust	The degree of trust older adults have in AI voice assistants, including reliability, safety, ease of use, human-centeredness, adaptability, and privacy protection	PTAI1	I believe the AI voice assistant can answer my questions and meet my needs more accurately and effectively	Kraus et al. (2024) and Le et al. (2024)
		PTAI2	I believe the AI voice assistant can better protect my privacy and personal information from being leaked or misused	
		PTAI3	I believe the AI voice assistant has more human-like characteristics such as friendliness, care, and patience	
Perceived AI experience	The perceived intelligence and service convenience level during human-AI interaction when older adults use AI voice assistants	PEAI1	I believe the AI voice assistant understands my speech and accent well, providing a smooth interaction experience	Balasubramaniam et al. (2023) and Jnr (2024)
		PEAI2	I believe the AI voice assistant provides accurate and relevant responses to my queries	
		PEAI3	I believe the AI voice assistant responds quickly, improving the overall interaction experience	
		PEAI4	I believe the AI voice assistant can be personalized based on my preferences, such as speech rate, language, and volume	
		PEAI5	I believe the AI voice assistant offers a seamless interaction with simple commands and convenient operations	
Facilitating conditions	Environmental and infrastructural factors that support older adults in adopting and using AI voice assistants	FC1	My home has sufficient technical infrastructure such as internet and hardware to support AI voice assistant use	Khechine et al. (2016) and Yu and Chen (2024)
		FC2	I can easily obtain and use the voice assistant, including purchase, installation, and maintenance	
		FC3	I believe there is sufficient technical support for using the AI voice assistant, such as customer service and training	
		FC4	I have support from family, friends, or community staff that helps me adopt and use AI voice assistants	
Performance expectancy	Older adults’ perception and expectation that AI voice assistants help complete daily tasks, offer convenience, and improve quality of life	PE1	I believe the AI voice assistant helps me complete daily tasks such as setting alarms, checking weather, and playing music	Khechine et al. (2016), Rouidi et al. (2022), Yu and Chen (2024)
		PE2	I believe the AI voice assistant helps me obtain necessary information quickly, such as news, addresses, and traffic	
		PE3	I believe the AI voice assistant helps me stay in touch with family and friends through messaging or video calls	
		PE4	I believe the AI voice assistant helps me manage my health, such as reminding medication or monitoring health data	
		PE5	I believe the AI voice assistant offers more entertainment and leisure options such as music, stories, and games	
Effort expectancy	Older adults’ perception and expectation of the learning cost, operational difficulty, and required skills for using AI voice assistants	EE1	It is easy for me to learn how to use the AI voice assistant, with minimal effort required to understand its basic functionalities	Khechine et al. (2016), Rouidi et al. (2022), Yu and Chen (2024)
		EE2	I find it easy to learn how to use the basic functions of the AI voice assistant	
		EE3	I feel confident in my ability to learn how to use the AI voice assistant, including its basic commands and features	
		EE4	Using the AI voice assistant is not a challenge for me	
Social influence	The extent to which older adults’ decision to adopt and use AI voice assistants is influenced by family, friends, and community	SI1	The opinions and experiences of my family and friends influence my decision to use AI voice assistants	Venkatesh (2022), Yu and Chen (2024)
		SI2	I perceive individuals who use AI voice assistants as more technologically capable	
		SI3	I am influenced by media and advertising promoting AI voice assistants	
		SI4	Using AI voice assistants is a trend. I want to keep up with the times, so I will learn to use them	
Intention to use AI voice assistant	The degree of willingness, attitude, and determination of older adults to use AI voice assistants	ITUAVA1	I think using AI voice assistants is a good idea	Gedrimiene et al. (2023)
		ITUAVA2	I think AI voice assistants make life more convenient	
		ITUAVA3	I think AI voice assistants are very valuable	
		ITUAVA4	I think AI voice assistants can replace traditional human services or other smart devices	
Usage behavior	The actual behaviors of older adults when using AI voice assistants	UB1	I make good use of AI voice assistants and am willing to continue learning new functions and skills	Gedrimiene et al. (2023), Le et al. (2024)
		UB2	I use AI voice assistants for relatively long durations	
		UB3	I use AI voice assistants for different types of tasks and functions	
		UB4	Using AI voice assistants has achieved the expected effect and improved my quality of life and convenience	

### 3.3 Experimental design

This study first conducted a pilot test from February 1 to February 5, 2025, with 30 elderly participants aged 60 and above. The purpose of the pilot test was to assess the comprehensibility of the questionnaire and the effectiveness of the research procedures. Based on the evaluation results, the research team revised ambiguous or inappropriate items in the questionnaire and made changes to the original items. For example, the original question “Learning to use the AI voice assistant requires some effort.” was vague, leading to difficulty in understanding for some respondents. Therefore, it was revised to “It is easy for me to learn how to use the AI voice assistant, with minimal effort required to understand its basic functionalities” to ensure the question was clearer and more understandable. In addition, some options were simplified to avoid redundant and repetitive descriptions. For instance, the original version of PE3 was “I am of the opinion that the AI voice assistant plays a significant role in facilitating communication with my family and friends, allowing me to maintain regular contact through various means such as messaging and video calls, thus bridging the gap in long-distance interactions.”

Before the formal study began, all participants underwent a health assessment to ensure they met the physical conditions for participation. The health assessments were carried out by community medical personnel appointed by the local public hospital, following standard physical examination protocols, in order to exclude elderly individuals with severe cardiovascular, respiratory, or neurological diseases, complications of diabetes, kidney failure, or other related conditions. Participants were informed during the screening process that they could withdraw from the study at any time without affecting their rights or benefits. All withdrawals were documented in accordance with the study protocol, ensuring the proper and transparent handling of withdrawals.

To ensure that illiterate participants could complete the questionnaire smoothly, the research team provided volunteer assistance. All volunteers were specially trained to ensure they understood the questionnaire content and could accurately convey it to illiterate participants. Volunteers used a standardized script to explain each item of the questionnaire to ensure that every illiterate participant fully understood the questions and options. To minimize bias in the translation and explanation process, all translated materials and explanatory scripts were verified and revised multiple times to ensure accuracy and consistency. The final survey tools and revision logs have been included in the appendix, and the volunteer training manual provides detailed guidance to ensure participants can successfully complete the questionnaire survey.

A total of 413 valid responses were collected. The demographic information of the participants is shown in Table 2. Among them, 53.1% were male, and 46.9% were female. In terms of age distribution, the majority of participants were aged 65–69 (42.6%), followed by those aged 70–74 (25.4%), 75 and above (16.2%), and 60–64 (15.7%). Regarding education levels, most participants had completed junior high school (35.4%) or high school (32.7%), while 19.9% had completed only primary school, and 8.5% had a college education. Notably, 3.6% of the participants were identified as illiterate. To address this, trained researchers and community volunteers assisted illiterate participants during the survey to ensure their full understanding and successful completion of the questionnaire.

**Table 2:** Demographic information (*N* = 413).

Category	Demographic characteristics	Number of participants	Percentage (%)
Gender	Male	219	53.1
	Female	194	46.9
Age	60–64	65	15.7
	65–69	176	42.6
	70–74	105	25.4
	75+	67	16.2
Education	Illiterate	15	3.6
	Elementary school	82	19.9
	Middle school	146	35.4
	High school	135	32.7
	University	35	8.5

### 3.4 Data analysis method

In this study, Structural Equation Modeling (SEM) was employed to explore the causal relationships and influence paths among the variables. Data analysis was conducted using SPSS version 26.0 and AMOS version 26.0. AMOS 26.0 was used to construct and evaluate the SEM, and to perform model fit and path analysis.

## 4 Results

### 4.1 Descriptive statistics

Descriptive statistics were used to measure the levels of each variable, primarily through the mean and standard deviation. The mean reflects the central tendency of the data, while the standard deviation indicates the degree of dispersion. Maximum and minimum values represent the range of the data.

As shown in the Table 3, the absolute values of skewness are all less than 3, and the absolute values of kurtosis are all less than 10, indicating that the data approximately follow a normal distribution. Among the eight measured variables, Effort Expectancy scored the lowest (2.743), while Facilitating Conditions scored the highest (3.597). The mean scores of the remaining variables are all above the midpoint value of 3, suggesting that respondents generally held positive evaluations of most variables. However, the relatively low score for Effort Expectancy indicates that participants perceived the effort required to learn or use the technology to be minimal.

**Table 3:** Descriptive statistics.

Variable	*N*	Min	Max	Mean	Std. Dev	Skewness	Kurtosis
Perceived AI trust	413	1	5	3.274	0.894	−0.127	−0.717
Perceived AI experience	413	1	5	3.369	0.870	−0.216	−0.692
Facilitating condition	413	1	5	3.597	0.890	−0.377	−0.542
Performance expectancy	413	1	5	3.130	0.956	−0.099	−0.744
Effort expectancy	413	1	5	2.743	0.978	0.231	−0.412
Social influence	413	1	5	3.301	0.890	−0.136	−0.729
Intention to use AI voice assistant	413	1	5	3.447	0.871	−0.228	−0.819
Usage behavior	413	1	5	3.558	0.935	−0.302	−0.776

### 4.2 Reliability and validity analysis

#### 4.2.1 Reliability analysis

Reliability analysis, also referred to as internal consistency analysis, is used to evaluate the stability, consistency, and dependability of measurement results. To ensure the accuracy of the findings, a reliability test was performed on valid questionnaire data before conducting further analysis. In social science research, Cronbach’s Alpha coefficient is commonly used to assess reliability. Generally, a coefficient above 0.9 indicates excellent reliability; between 0.8 and 0.9 is considered very good; between 0.7 and 0.8 is acceptable; between 0.6 and 0.7 is marginally acceptable; and below 0.6 suggests the need for revision.

From the Table 4, it is evident that all Cronbach’s Alpha coefficients exceed 0.8, indicating that the data demonstrate good reliability. Regarding the “Alpha if Item Deleted,” the removal of any item does not significantly improve the overall reliability, suggesting that none of the items should be removed. As for the “Corrected Item-Total Correlation” (CITC), all CITC values are above 0.4, indicating strong inter-item correlations and good internal consistency. In summary, with all reliability coefficients above 0.8, the data demonstrate high reliability and are suitable for further analysis.

**Table 4:** Reliability analysis results.

Variable	Item	Corrected item-total correlation	Cronbach’s alpha if item deleted	Reliability
Perceived AI trust	PTAI1	0.71	0.79	0.840
	PTAI2	0.617	0.815	
	PTAI3	0.625	0.813	
	PTAI4	0.604	0.819	
	PTAI5	0.668	0.802	
Perceived AI experience	PEAI1	0.654	0.853	0.87
	PEAI2	0.738	0.832	
	PEAI3	0.673	0.849	
	PEAI4	0.712	0.839	
	PEAI5	0.702	0.842	
Facilitating condition	FC1	0.718	0.854	0.88
	FC2	0.722	0.853	
	FC3	0.767	0.835	
	FC4	0.754	0.84	
Performance expectancy	PE1	0.752	0.84	0.877
	PE2	0.697	0.853	
	PE3	0.671	0.86	
	PE4	0.713	0.85	
	PE5	0.712	0.85	
Effort expectancy	EE1	0.685	0.791	0.839
	EE2	0.669	0.797	
	EE3	0.678	0.793	
	EE4	0.654	0.804	
Social influence	SI1	0.711	0.813	0.857
	SI2	0.664	0.832	
	SI3	0.688	0.823	
	SI4	0.739	0.801	
Intention to use AI voice assistant	ITUAVA1	0.745	0.807	0.861
	ITUAVA2	0.695	0.829	
	ITUAVA3	0.71	0.822	
	ITUAVA4	0.686	0.833	
Usage behavior	UB1	0.803	0.851	0.895
	UB2	0.737	0.877	
	UB3	0.761	0.867	
	UB4	0.772	0.863	

#### 4.2.2 Validity analysis

Validity refers to the extent to which a test or scale accurately measures the psychological or behavioral traits it is intended to measure—that is, the accuracy and credibility of the results. Generally, a lower significance level (*p* < 0.05) in Bartlett’s Test of Sphericity indicates that meaningful relationships exist among the original variables. The KMO (Kaiser-Meyer-Olkin) value is used to assess the sampling adequacy by comparing simple and partial correlations among items, ranging between 0 and 1. The thresholds for factor analysis suitability are: above 0.9 = excellent; 0.7–0.9 = suitable; 0.6–0.7 = moderately suitable; 0.5–0.6 = marginally unsuitable; below 0.5 = not suitable.

As shown in the Table 5, the KMO value is 0.903, which exceeds the 0.8 threshold, indicating good construct validity and that the data are highly suitable for factor extraction.

**Table 5:** KMO and Bartlett’s test.

KMO measure of sampling adequacy		0.903
Bartlett’s test of Sphericity	Approx. Chi-Square	7545.594
	Degrees of freedom	595
	Significance	0.000

### 4.3 Bivariate correlation analysis

Correlation analysis describes and evaluates the nature and strength of the relationship between two or more variables. A correlation coefficient greater than 0 indicates a positive relationship between variables, while a coefficient less than 0 indicates a negative relationship.

From the Table 6, all variables show significant correlations with each other. Specifically, the correlation between perceived trust and usage behavior is 0.293 (*p* < 0.01), indicating a significant positive relationship. The correlation between perceived experience and usage behavior is 0.378 (*p* < 0.01), suggesting a strong positive influence of perceived experience on behavior. Facilitating conditions correlate with usage behavior at 0.324 (*p* < 0.01), indicating that improved facilitating conditions enhance usage behavior. Performance expectancy shows a positive correlation of 0.291 (*p* < 0.01) with usage behavior, confirming its significant role.

**Table 6:** Pearson correlation matrix.

Variable	PTAI	PEAI	FC	PE	EE	SI	IUAIVA	UB
PTAI	1							
PEAI	0.256**	1						
FC	0.266**	0.398**	1					
PE	0.303**	0.303**	0.300**	1				
EE	−0.210**	−0.198**	−0.215**	−0.223**	1			
SI	0.379**	0.295**	0.300**	0.328**	−0.187**	1		
IUAIVA	0.350**	0.322**	0.344**	0.340**	−0.205**	0.279**	1	
UB	0.293**	0.378**	0.324**	0.291**	−0.192**	0.286**	0.322**	1

*Correlation is significant at the 0.01 level (2-tailed).

Notably, effort expectancy has a negative correlation of −0.192 (*p* < 0.05) with usage behavior, suggesting that higher effort expectancy may reduce actual usage, which aligns with the hypothesized negative relationship. Social influence correlates positively with usage behavior at 0.286 (*p* < 0.01), highlighting its promotive effect. Intention to use correlates with usage behavior at 0.322 (*p* < 0.01), indicating that increased willingness leads to more active usage.

### 4.4 Confirmatory factor analysis

**Figure 3:**
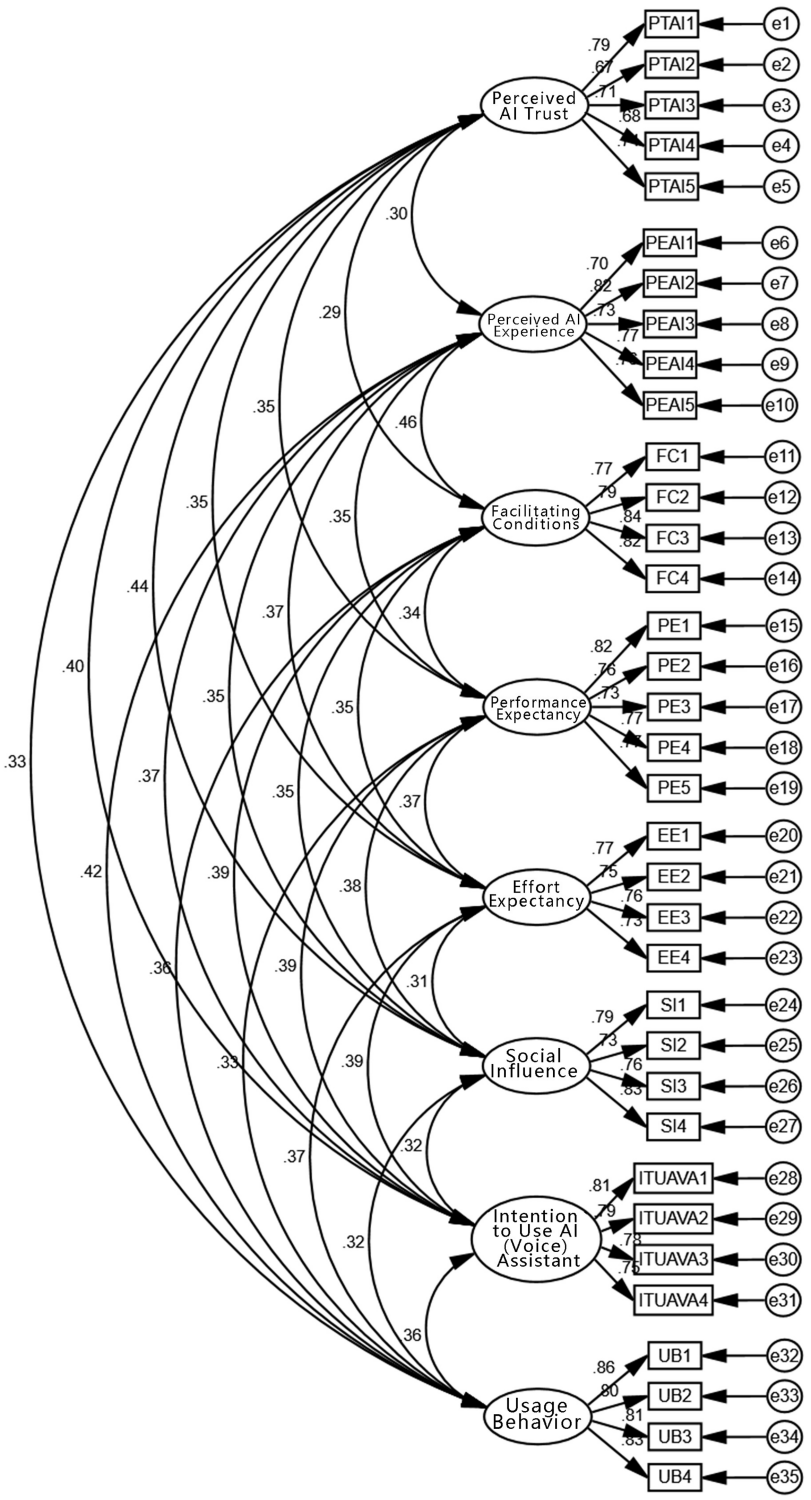
Confirmatory factor analysis (CFA) path diagram of the measurement model.

As shown in Figure 3, the path diagram presents the standardized factor loadings obtained from the Confirmatory Factor Analysis (CFA). Each latent construct (e.g., Perceived Trust in AI, Perceived AI Experience) is measured by multiple observed variables, and the model demonstrates good convergent and discriminant validity. The CFA results confirm the measurement model’s structure and provide empirical support for the reliability and validity of the latent constructs.

**Figure 4:**
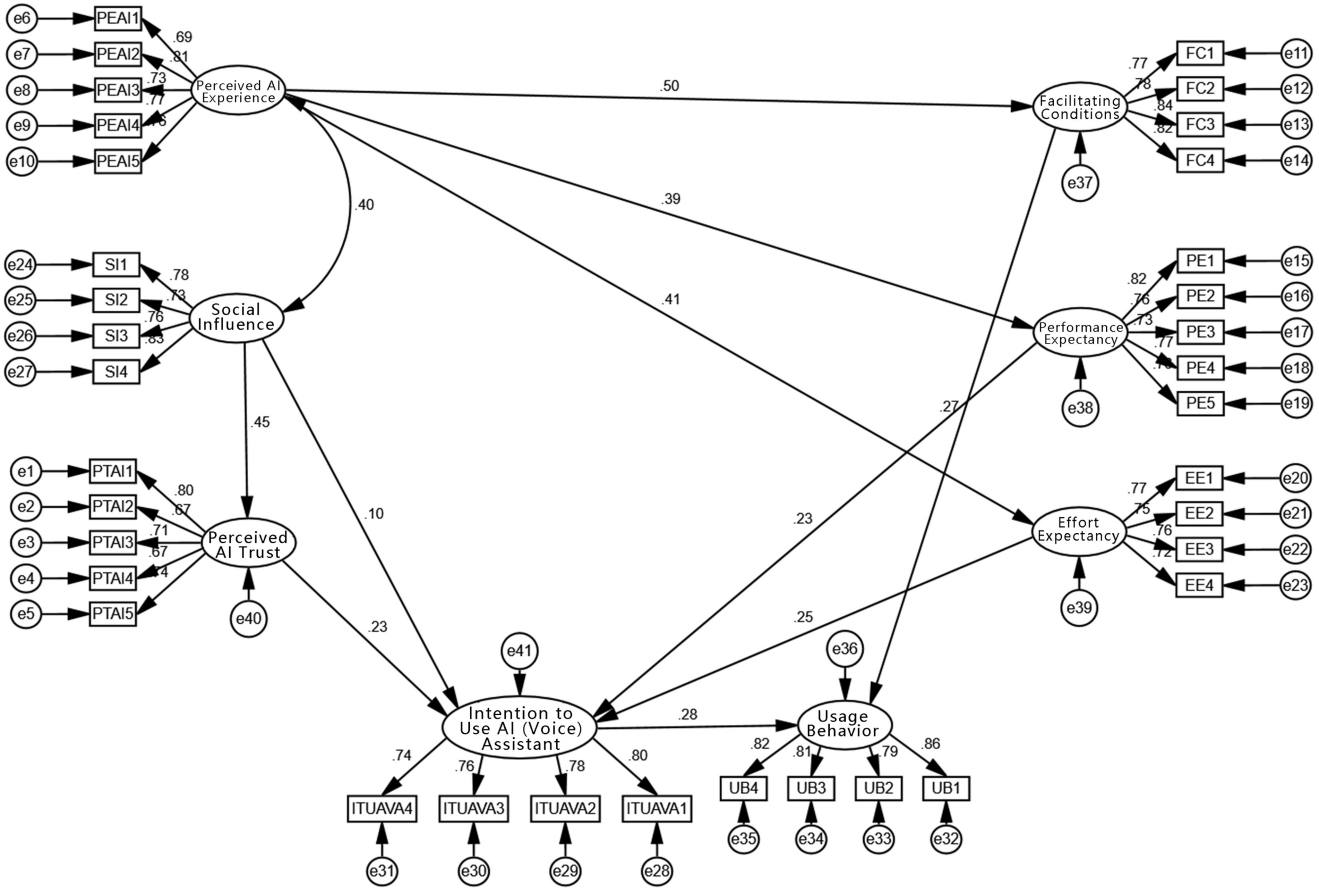
Path coefficient diagram.

As shown in the Table 7, the CMIN/DF value is 1.144, which is less than 3; RMSEA is 0.019, below the threshold of 0.08, indicating a good fit. GFI (0.925), IFI (0.989), CFI (0.989), RFI (0.913), and NFI (0.922) all exceed the recommended 0.90 threshold. PNFI is 0.824, which is greater than 0.50. All goodness-of-fit indices meet standard benchmarks, indicating that the model fits well (Figure 4).

**Table 7:** Model fit indices.

Common indices	Criteria	Statistic	Fit evaluation
CMIN	–	608.759	–
DF	–	532	–
CMIN/DF	<3	1.144	Good
RMSEA	<0.08	0.019	Good
GFI	>0.90	0.925	Good
IFI	>0.90	0.989	Good
CFI	>0.90	0.989	Good
RFI	>0.90	0.913	Good
NFI	>0.90	0.922	Good
PNFI	>0.50	0.824	Good

The standardized factor loading table shows the strength of the relationships between latent factors and their corresponding measurement items. All items are significant at the 0.001 level (*p* < 0.001), and all standardized loadings exceed 0.6, indicating strong factor-item relationships and good convergent validity (see Table 8).

**Table 8:** Standardized factor loadings.

Latent variable	Item	Std. loading	Std. error	*z*	*p*	Loading coefficient
Perceived AI trust	PTAI1	1.000				0.792
	PTAI2	0.875	0.066	13.346	***	0.672
	PTAI3	0.948	0.067	14.184	***	0.710
	PTAI4	0.880	0.065	13.455	***	0.677
	PTAI5	0.907	0.061	14.769	***	0.738
Perceived AI experience	PEAI1	1.000				0.701
	PEAI2	1.268	0.085	14.860	***	0.816
	PEAI3	1.054	0.078	13.508	***	0.732
	PEAI4	1.221	0.086	14.199	***	0.774
	PEAI5	1.163	0.083	13.992	***	0.761
	FC1	1.000				0.768
	FC2	1.111	0.069	16.174	***	0.787
Facilitating condition	FC3	1.209	0.070	17.310	***	0.840
	FC4	1.105	0.065	16.966	***	0.823
	PE1	1.000				0.818
	PE2	0.927	0.056	16.544	***	0.760
Performance expectancy	PE3	0.903	0.058	15.628	***	0.726
	PE4	0.858	0.051	16.824	***	0.770
	PE5	0.836	0.050	16.760	***	0.768
	EE1	1.000				0.773
Effort expectancy	EE2	0.994	0.069	14.449	***	0.747
	EE3	1.033	0.070	14.670	***	0.759
	EE4	0.986	0.070	14.149	***	0.731
	SI1	1.000				0.786
	SI2	0.854	0.058	14.725	***	0.726
Social influence	SI3	0.942	0.061	15.419	***	0.758
	SI4	1.070	0.064	16.800	***	0.827
	ITUAVA1	1.000				0.812
Intention to use AI voice assistant	ITUAVA2	0.993	0.060	16.677	***	0.787
	ITUAVA3	0.903	0.055	16.414	***	0.776
	ITUAVA4	0.826	0.052	15.780	***	0.749
Usage behavior	UB1	1.000				0.863
	UB2	0.985	0.051	19.347	***	0.800
	UB3	0.921	0.046	19.893	***	0.815
	UB4	0.924	0.045	20.361	***	0.828

CR (Construct Reliability) is calculated using factor loadings and represents internal consistency. Values above 0.7 are considered acceptable. AVE (Average Variance Extracted) represents the degree of convergent validity. Values above 0.5 are generally acceptable. In this study, all CR values are above 0.7, and all AVE values exceed 0.5, indicating good convergent validity (see Table 9).

**Table 9:** Construct reliability and convergent validity.

Variable	CR	AVE
Perceived AI trust	0.842	0.517
Perceived AI experience	0.871	0.574
Facilitating condition	0.880	0.648
Performance expectancy	0.878	0.591
Effort expectancy	0.839	0.566
Social influence	0.857	0.601
Intention to use AI voice assistant	0.862	0.610
Usage behavior	0.896	0.684

All diagonal values (square roots of AVE) exceed the corresponding inter-construct correlation coefficients in their rows and columns, confirming good discriminant validity (see Table 10).

**Table 10:** Discriminant validity.

Variable	PTAI	PEAI	FC	PE	EE	SI	IUAVA	UB
PTAI	**0.719**							
PEAI	0.256**	**0.758**						
FC	0.266**	0.398**	**0.805**					
PE	0.303**	0.303**	0.300**	**0.769**				
EE	−0.210**	−0.198**	−0.215**	−0.223**	**0.690**			
SI	0.379**	0.295**	0.300**	0.328**	−0.187**	**0.775**		
IUAVA	0.350**	0.322**	0.344**	0.340**	−0.205**	0.279**	**0.781**	
UB	0.293**	0.378**	0.324**	0.291**	−0.192**	0.286**	0.322**	**0.827**

*Bolded diagonal values represent the square root of AVE.

### 4.5 Hypothesis testing

As shown in Table 11, perceived AI experience significantly influenced performance expectancy, effort expectancy, and facilitating conditions. The standardized path coefficient from perceived AI experience to performance expectancy was 0.393, with a z-value of 6.785 (*p* < 0.05), indicating that a more positive user experience with AI voice assistants leads to higher performance expectations among older adults. Thus, H5-a is supported.

**Table 11:** Hypothesis path analysis.

Path			Estimate	S. E.	C. R.	*p*	Estimate	Hypothesis
Perceived AI experience	→	Performance expectancy	0.556	0.082	6.785	***	0.393	Supported
Perceived AI experience	→	Effort expectancy	−0.218	0.067	−3.254	***	−0.192	Supported
Perceived AI experience	→	Facilitating condition	0.538	0.065	8.273	***	0.499	Supported
Social influence	→	Perceived AI trust	0.478	0.062	7.76	***	0.454	Supported
Performance expectancy	→	Intention to use AI voice assistant	0.194	0.045	4.261	***	0.229	Supported
Effort expectancy	→	Intention to use AI voice assistant	−0.167	0.058	−2.879	0.004	−0.145	Supported
Facilitating condition	→	Intention to use AI voice assistant	0.325	0.065	5.035	***	0.27	Supported
Social influence	→	Intention to use AI voice assistant	0.096	0.061	1.589	0.112	0.098	Not supported
Social influence	→	Perceived AI trust	0.478	0.062	7.76	***	0.454	Supported
Perceived AI trust	→	Intention to use AI voice assistant	0.216	0.058	3.752	***	0.232	Supported
Intention to use AI voice assistant	→	Usage behavior	0.306	0.059	5.165	***	0.281	Supported

Perceived AI experience also had a significant negative effect on effort expectancy (β = −0.192, *z* = −3.254, *p* < 0.05), suggesting that a better experience reduces perceived effort in learning and using AI assistants. H5-b is supported. Additionally, the effect of perceived AI experience on facilitating conditions was significant and positive (β = 0.499, *z* = 8.273, *p* < 0.05), meaning that when AI experience is favorable, older adults are more likely to perceive external support (e.g., device availability, software usability, training resources) as adequate. Thus, H5-c is supported.

Next, social influence had a significant positive effect on perceived trust in AI (β = 0.454, *z* = 7.760, *p* < 0.05), indicating that when the social environment (e.g., family, friends, community) is supportive of AI voice assistants, older adults tend to trust the technology more. Therefore, H6 is supported. However, the effect of social influence on intention to use was not statistically significant (β = 0.098, *z* = 1.589, *p* > 0.05). This implies that while social influence can enhance trust, it does not necessarily translate into stronger usage intention—older adults are more likely to rely on personal experience and perceived utility. H3 is not supported.

Among the factors influencing intention to use, performance expectancy had a significant positive effect (β = 0.229, *z* = 4.261, *p* < 0.05), indicating that when older adults perceive high functional value in AI assistants, their intention to use them increases. H1 is supported. Effort expectancy negatively influenced intention to use (β = −0.145, *z* = −2.879, *p* < 0.05), meaning higher perceived difficulty lowers willingness. H2 is supported. Facilitating conditions also positively influenced usage intention (β = 0.270, *z* = 5.035, *p* < 0.05), suggesting that a more supportive environment (e.g., technical infrastructure, learning resources) boosts willingness to use AI assistants. H4 is supported.

Additionally, perceived trust in AI significantly influenced intention to use (β = 0.232, *z* = 3.752, *p* < 0.05). This implies that when older adults trust AI voice assistants (e.g., due to privacy protection, accurate recognition, and friendly interaction), their intention to use increases. H7 is supported.

Finally, intention to use significantly affected actual usage behavior (β = 0.281, *z* = 5.165, *p* < 0.05), indicating that stronger willingness leads to higher likelihood of translating intention into action. H8 is supported.

## 5 Discussion

### 5.1 General discussion

This study aimed to investigate the key factors influencing older adults’ adoption of AI voice assistants by extending the Unified Theory of Acceptance and Use of Technology (UTAUT), focusing on a sample of older adults from two retirement communities in Shanxi Province. It examined the roles of perceived AI experience, perceived trust, performance expectancy, and social influence in shaping older adults’ behavioral intentions toward technology adoption.

Overall, perceived AI experience, performance expectancy, facilitating conditions, and perceived trust in AI were positively correlated with older adults’ intention to use AI voice assistants, whereas effort expectancy was negatively correlated with usage intention. Moreover, the positive correlation between intention to use and actual usage behavior was confirmed. However, social influence did not have a significant impact on usage intention, suggesting that older adults in our sample may prioritize their own technological experiences and usability perceptions over external social encouragement when deciding whether to adopt AI voice assistants. These findings are based on a sample from two retirement communities in Shanxi Province, and future studies should explore whether these results hold in other cultural and demographic contexts.

Specifically, Perceived AI experience is positively correlated with performance expectancy and facilitating conditions, while it is negatively correlated with effort expectancy. This indicates that when older adults have better interactions with AI voice assistants, they are more likely to expect functional benefits and perceive lower difficulty in learning and using the technology. In other words, a positive AI experience not only strengthens users’ trust but also alleviates concerns about the costs of adoption, thereby enhancing both intention and actual usage. This finding aligns with Venkatesh et al.’s (2012) UTAUT framework and further supports the pivotal role of user experience in technology acceptance.

In addition, the positive effects of performance expectancy and facilitating conditions on usage intention were confirmed. When older adults believe that AI voice assistants are useful and supported by external infrastructure (e.g., device compatibility, training), they are more inclined to adopt the technology. Notably, the negative impact of effort expectancy on usage intention was also significant, suggesting that when older adults perceive high learning costs, they are more likely to reject the technology. This finding is consistent with Davis’s (1989) theory of perceived ease of use, highlighting the importance of reducing learning barriers to improve older adults’ acceptance. Hence, developers should focus on simplifying operations, optimizing interaction design, and offering easy-to-understand learning resources to lower the entry barrier for older users.

Perceived trust in AI also had a significant positive impact on usage intention. When older adults perceive AI voice assistants as trustworthy—ensuring privacy, recognition accuracy, and safe interactions—they are more willing to try the technology. This finding echoes research by Gefen et al. (2003), further reinforcing trust as a central factor in technology adoption.

However, social influence did not have a significant impact on usage intention, which contrasts with some previous studies on technology acceptance. One possible explanation is that older adults may place more importance on their own technological experiences and perceptions of usability than on external social endorsements. This is consistent with findings in previous literature, which indicate that older adults are often more skeptical of external influences when making decisions about technology adoption (Karkera et al., 2023). Moreover, older adults may be particularly sensitive to privacy concerns and the perceived risks associated with data security, which could dampen the effect of social influence, even when social support is present (Boström et al., 2022). In addition, the complexity and perceived difficulty of using technology may undermine the role of social influence. Older adults may be less receptive to social influence if the technology is not intuitive or if they perceive significant barriers to learning and usage (Karkera et al., 2023). Furthermore, previous studies have shown that older users may be more influenced by trust in the technology itself rather than external social factors (Gefen et al., 2003). Therefore, while social influence can foster trust in technology, it may not be as effective in directly promoting adoption when the technology’s perceived risks and usability concerns are high. These findings suggest the importance of addressing older adults’ privacy concerns and reducing the perceived complexity of AI voice assistants to enhance adoption.

Finally, the study confirmed the positive relationship between intention to use and actual usage behavior. When older adults are willing to use AI voice assistants, they are more likely to convert this intention into actual behavior. This result aligns with Ajzen’s (1991) Theory of Planned Behavior (TPB), reaffirming the pivotal role of intention in behavior formation. Therefore, enhancing users’ intention is crucial for increasing actual usage of AI voice assistants among older adults.

### 5.2 Theoretical contributions

This study provides in-depth theoretical insights into older adults’ technology adoption behavior and extends the UTAUT model. First, the applicability of the UTAUT model among older adults was confirmed, especially the influence of performance expectancy, effort expectancy, facilitating conditions, and perceived trust in AI on their intention to use AI voice assistants. Moreover, this study introduced perceived AI experience as a new variable, broadening the UTAUT framework and highlighting the critical role of user interaction experience in technology adoption.

Second, the study emphasized the central role of trust in the technology acceptance process among older adults. It confirmed the significant impact of perceived trust in AI on usage intention. This finding supports the insights of McKnight et al. (2002) and Jarvenpaa et al. (2000) on the importance of trust in technology adoption and sheds light on how older adults assess the safety, stability, and reliability of AI voice assistants based on trust factors.

In addition, the study found that social influence had no significant effect on older adults’ intention to use AI voice assistants. This result differs from some previous findings and suggests that adoption among older adults is primarily driven by personal experience rather than social encouragement. This challenges the universality of the social influence construct in the UTAUT model and calls for future research to explore the mechanisms of social influence across different user groups.

### 5.3 Practical implications

The findings of this study offer practical guidance for AI voice assistant developers, policymakers, and community organizations.

For technology developers, the results indicate that optimizing the AI interaction experience is critical to enhancing older adults’ willingness to use the technology. Therefore, AI voice assistants should be designed with age-friendly interfaces, simplified procedures, voice-guided functions, and clearly segmented tasks to reduce cognitive load and learning costs. To strengthen trust, developers should also enhance data security and privacy protections by offering controllable data access, improving voice recognition accuracy, and minimizing system errors.

For policymakers and community organizations, the study highlights effort expectancy as a major barrier to adoption—older adults often perceive the learning curve as too steep. Governments and institutions can respond by organizing digital literacy training, providing usage guides in community centers or senior colleges, and offering volunteer or family support to help older adults become familiar with the technology. Additionally, governments can promote age-inclusive smart device designs and encourage the development of AI products tailored to older users, reducing the digital divide.

### 5.4 Limitations and future research directions

Despite its contributions, this study has several limitations that should be addressed in future research. First, the data were collected from a specific geographic area, and the sample may exhibit biases in terms of age, education level, and technical proficiency. This limits the generalizability of the findings. Future research should expand to include older adults from diverse cultural and social backgrounds to enable cross-cultural comparisons.

Second, the study employed a cross-sectional design, which cannot capture the dynamic nature of technology acceptance. Since adoption is a long-term process, older adults’ attitudes and behaviors may evolve as their proficiency increases or as the technology improves. Future research should adopt longitudinal designs to track changes over time and better understand the long-term mechanisms of adoption.

Third, while the study focused on perceived AI experience, trust, and other cognitive factors, it did not explore additional influences such as emotional attachment, health status, financial capacity, and social support. Future work could integrate these psychosocial variables to develop a more comprehensive model of older adults’ technology acceptance.

In terms of methodology, this study relied primarily on self-report data, which may be subject to social desirability bias or subjective distortion. Although anonymity helped mitigate some of this bias, objective behavioral data—such as AI usage logs—should be incorporated in future studies. A mixed-methods approach involving interviews, experiments, and behavioral tracking could provide deeper insights into usage patterns and psychological mechanisms.

Finally, the non-significant impact of social influence raises questions about its role in older adults’ technology acceptance. Future research could explore the differential effects of social influence sources (e.g., family, peers, media) and examine how these factors affect trust and intention in different contexts. It may also be useful to study how social interactions foster trust, and how that trust subsequently influences intention and behavior.

In conclusion, future research should expand sample diversity, adopt longitudinal and mixed-methods approaches, and integrate broader social and psychological factors. This will not only enhance theoretical understanding of technology acceptance but also inform more targeted strategies to promote AI adoption among older adults.

## 6 Conclusion

This study extends the Unified Theory of Acceptance and Use of Technology (UTAUT) to explore the impact of factors such as perceived AI experience and perceived AI trustworthiness on older adults’ adoption of AI voice assistants. The results indicate that performance expectancy, facilitating conditions, perceived AI trustworthiness, and perceived AI experience all have a significant positive effect on older adults’ intention to use AI voice assistants, while effort expectancy has a negative impact. Although social influence significantly affects perceived AI trustworthiness, it does not have a direct impact on intention to use.

This study provides new insights into understanding older adults’ adoption of AI technologies, particularly in terms of how perceived AI experience and perceived AI trustworthiness influence technology adoption. The findings not only enrich the application of the UTAUT model in older adult populations but also offer practical guidance for developing age-friendly AI voice assistants. Future research could further explore the influence of other psychosocial factors on older adults’ technology adoption, and adopt longitudinal and mixed-methods approaches to track long-term changes and psychological mechanisms in AI voice assistant adoption.

## Data Availability

The raw data supporting the conclusions of this article will be made available by the authors, without undue reservation.
